# Defining One Health: Environment versus ecosystem

**DOI:** 10.1079/cabionehealth.2026.0009

**Published:** 2026-05-20

**Authors:** Andrea Ford, Lisa Boden, Paulo Ferinho, Saana Jukola, Lucy Robertson, Saskia Stucki, Jakob Zinsstag

**Affiliations:** 1 Centre for Biomedicine, Self and Society, Usher Building, The University of Edinburgh, –7 Little France Road, Edinburgh EH16 4UX, UK; 2 Royal (Dick) School of Veterinary Studies, Easter Bush Campus, Roslin, EH25 9RG, UK; 3 Global Health and Tropical Medicine, Instituto de Higiene e Medicina Tropical, Universidade Nova de Lisboa, 100 Rua da Junqueira, Alcantara, Lisbon, Portugal; 4 Philosophy Section, University of Twente, Drienerlolaan 5, 7522 NB Enschede, The Netherlands; 5 Department of Paraclinical Sciences, Faculty of Veterinary Medicine, Norwegian University of Life Sciences, Elizabeth Stephansens vei 15, 1433 Ås, Norway; 6 School of Management and Law, Zurich University of Applied Sciences, Gertrudstrasse 15, 8401 Winterthur, Switzerland; 7 Swiss Tropical and Public Health Institute, Kreuzstr. 2, 4123 Allschwil, Switzerland; 8 University of Basel, Petersplatz 1, 4003 Basel, Switzerland

**Keywords:** environment, ecosystem, philosophy, epistemology and ontology, balance, integration, OHHLEP definition

## Abstract

**Background:**

This article seeks greater precision in defining One Health by clarifying ambiguities and elaborating issues that arise from the One Health High-Level Expert Panel (OHHLEP) definition, while acknowledging the utility of the OHHLEP definition and the consensus it represents. In particular, we clarify certain implications of how the third, environmental component of One Health is understood.

**Methods:**

We developed our argument by taking part in the interdisciplinary Science Advice for Policy by European Academies (SAPEA) consortium for One Health Governance in the European Union. We are a highly interdisciplinary team that has developed this theoretical position by meeting throughout 2024 to write the report chapter on which it is based.

**Results:**

We suggest that whereas an “environment” implies something external (and perhaps subordinate) to humans and animals, an “ecosystem” implies a system of which both humans and animals are part (alongside plants, chemicals, microbes, etc.). We explore three sets of concepts within the OHHLEP definition: “integrated” and “unifying”; “balanced” and “optimised”; “closely linked”; and “interdependent.” Lastly, we explain our synergies and complementarity with adjacent concepts including “Planetary Health”.

**Conclusions:**

To us, clarity in these nuances seems key in the development of One Health away from an anthropocentric framing towards a more integrated and balanced framing. While we argue that the ecosystemic, holistic view is preferable and better aligned with One Health’s expanding scope, we note that conditions for making practical changes are not yet able to accommodate holism.

## Introduction

### Why Discuss The Definition of One Health?

Integrated approaches to health, like One Health, address the complex (or ‘wicked’) social, economic, and ecological problems affecting human, animal, and plant health that are embedded in the United Nations Sustainable Development Goals (Harris *et al*., 2010). Over the past two decades, as the One Health concept has evolved, the ways human health, animal health and the health of environments (including, but not limited to, plants) are intertwined have become more broadly recognised. Yet, this complex intertwining is difficult to fully grasp. Additionally, given how existing institutions are based around isolated components of One Health, it is also difficult to act on this complexity. Precision about our language can start to help with this problem.

In this article, we discuss the authoritative definition of One Health agreed by the One Health High Level Expert Panel (OHHLEP) in 2022 and suggest some areas where it could be more precise, particularly regarding the difference between an environment and an ecosystem. We emphatically acknowledge that the OHHLEP definition was a useful step forward; here, our focus is to try to refine certain nuances. This article evolved out of the first chapter of the SAPEA One Health governance in the European Union report prepared by a SAPEA (Science Advice for Policy by European Academies) consortium, as part of the Scientific Advice Mechanism of The European Commission (Group of Chief Scientific Advisors, 2024). It is worth noting that to ensure alignment with the existing consensus our suggestions were not adopted by the Group of Chief Scientific Advisors to the European Commission. The importance of our contribution is, to a large extent, the nuanced discussion of the consensualised definition, which is what we share here.

The term ‘One Health’ was introduced to describe interventions that include closer cooperation between human and animal health, and any other sector that, when compared to single-sector approaches, provides an incremental benefit (i.e., added value through more economically and logistically efficient ways of safeguarding and promoting health) (Zinsstag *et al*., 2005). While paradigms like “environmental health” and “planetary health” focus on human health and prioritise human interests, One Health is distinguished by its potential to move away from such anthropocentrism (theoretically at least). Therefore, a story of what One Health *is* and what it *can be* underlies this article.

Hence, we engage with concepts that de-centre humans and point towards interconnection and interdependence, which should facilitate operationalisation and optimisation of the One Health approach. We acknowledge that any definition needs sufficient flexibility for One Health to be operationalised in practice, allowing for policy clarification, strategy development, prioritisation, and efficient progress away from anthropocentrism and towards holistic ecological health, yet starting from within existing paradigms. This contrasts with many initiatives labelled as “One Health”, which have implemented programmes with a desired outcome (such as cost-benefit optimisation), but without a rigorous underpinning theoretical framework. The term “One Health” has been used widely and sometimes rather loosely. Extra precision in the definition allows us to identify those elements of the complex problem-scape that are being addressed, and provides a common language that facilitates interdisciplinary and transdisciplinary collaboration.

We are working from the European Union (EU) context yet also consider interconnections and interdependencies from a global perspective; while operationalising European policy, we must build capacity in other regions, tackling what is both a global issue and local concern.

## Methods

### Starting From Ohhlep and Clarifying Our Terms

We acknowledge and commend the definition from OHHLEP. Working from it, as we do, facilitates adaptation to the One Health Joint Plan of Action from the Quadripartite Organisations (FAO, UNEP, WHO and WOAH) (OHHLEP *et al*., 2022). “Acquired language” is an accepted best practice for professional working groups; it entails adopting language used in the past, unless it is uncomfortable for some reason, to acknowledge that ideas have been previously discussed and consensualised.

One Health is an integrated, unifying approach that aims to sustainably balance and optimise the health of people, animals, and ecosystems. It recognises the health of humans, domestic and wild animals, plants, and the wider environment (including ecosystems) are closely linked and interdependent (OHHLEP et al., 2022).

This is a usefully all-encompassing definition. To develop a theoretical foundation, aspects of it require some scrutiny. Notably, the differences between “environments” and “ecosystems” need to be elaborated. The definition also lacks clarity on the adopted definition for “health” and whether this differs for people, animals, environments, and ecosystems. Furthermore, important philosophical concepts and their relations remain unclear, namely:

- “integrated” and “unifying”;
- “balanced” and “optimised”;
- “closely linked” and “interdependent”.

Our starting point is that clarity is needed about the theoretical foundations of One Health, with attention to the way knowledge is framed, generated, and evaluated (our epistemology), particularly in a multi-cultural context. This has implications for our understanding of reality (our ontology), shifting from a focus on humans (anthropocentrism) to one of social-ecological systems. The way we define One Health shapes our governance structures, methods, research approaches and topics, and the indicators we use to monitor One Health operationalisation. Without addressing the issues explicitly, in our approach we also take into consideration:

- the need to move towards *interdisciplinary and transdisciplinary* collaboration in research, policy, and implementation;
- the importance of *sociocultural considerations*;
- the importance of *economic considerations*.

An elaborated discussion of these elements can be found in the first chapter of the SAPEA One Health governance in the European Union report (Group of Chief Scientific Advisors, 2024). By shedding light on these elements, we intend to enhance the acceptability, impact and operability of the definition, and ensure sustainability.

## Results and Discussion

### Shifting The Focus From Anthropocentrism

We begin with a philosophical grounding that reflects the diverse expertise of the authors. Our approach can be contrasted with traditional approaches to studying and promoting health. Clinical medicine, including veterinary medicine, tends to consider the health of the patient in isolation from the health and wellbeing of other living organisms, and a dichotomy between a human or non-human animal organism and the environment is implicit. Public health (including veterinary public health) has maintained this anthropocentric focus, despite broadening the scope of the analysis from an individual organism to group and population levels. Epistemologically, our approach differs by positing that the conditions for the health of a living being cannot be properly analysed and understood without considering the health and wellbeing of other living beings and the ecosystems they constitute (e.g. Zinsstag *et al*., 2011). Ontologically, our approach questions the assumption that the health of an organism is independent of the health of other beings and the proper functioning of the ecosystem.

This is consistent with general, historical One Health principles that by taking a comprehensive view, encompassing the health and wellbeing of all living organisms and their habitats, we can better understand and address the complex interactions that contribute to overall ecosystem health and therefore to human wellbeing. This is an unconventional way of understanding the world. Our existing terms encourage more conventional thinking about entities instead of wholes or relationships. Because of this, we are taking this opportunity to be precise about our concepts and clarify some of the ambiguities implicit in the way One Health is often discussed. We recognise that epistemology (how things are known) and ontology (what things are) are inextricable and co-constituted, and therefore how we describe and frame the problem is of great importance.

### “Ecosystem” and “Environment”

By “ecosystem”, we mean a holistic system comprising living and non-living entities that are both interconnected and interdependent. It is important to remember that both humans and non-human animals are part of ecosystems (whereas humans are not usually considered part of environments, and often domestic animals are not either). Ecosystems, in turn, are plural and localised, but also interconnected with, and interdependent on, each other, so that our planet can also be described as an ecosystem. A related conceptual term is “social-ecological systems” (SES), which explicitly includes human actors in a systemic interaction with environments (or what are sometimes called “natural resources”) within ecosystems (McGinnis and Ostrom, 2014).

By “environment”, we mean what is part of an ecosystem, but not a human or non-human animal. A non-exhaustive list would include plants, microbes, fungi, soil, waterways, the atmosphere, manufactured materials and chemicals, and the climate. There is inherent and inevitable imprecision in such a list because we cannot separate some of these aspects, e.g. microbes living on/ in animals, but the conversations being had about these entities are relevant to One Health’s third component. We hesitate to mention some of these environmental components and not others (e.g. plants, which are mentioned specifically in the OHHLEP definition), as it can foreclose recognition of how multifaceted “environments” are. We also want to be clear that we include person-made environments and not just those that are “natural,” because so many of our environments are engineered (or exposed to consequences from other engineered spaces), and it no longer makes sense to think of these as distinct.

Where possible, we avoid the term “environment”, as it perpetuates the idea that an organism (or sometimes a species) is the “figure” on an environmental “background” or surrounded by an environmental “context”, that the environment is a place instead of an entity or collective of entities in its own right. (For similar reasons, we also avoid the term “nature”, which usually implies places and processes separated from human activity.) By contrast, an ecological view emphasises complex connections and dependencies between living and non-living entities. We also avoid the term “environmental health”, as this refers to a specific, established field within public health dealing with aspects of human health, including quality of life, determined by physical, chemical, biological, social and psychosocial factors in the environment (Santos *et al*., 2019).

It is important to note that our definitions of “ecosystem” and “environment” are not necessarily implied in the OHHLEP definition of One Health, which lists “ecosystems” as a third element after humans and animals (OHHLEP *et al*., 2022). This could be read as suggesting that humans and animals are not part of ecosystems, or that humans are not part of animals (some advocate that the triad be conceived of as concentric circles, for that reason). The OHHLEP definition also mentions ecosystems as “included in” wider environments, whereas “ecosystem” is the more inclusive term as we define it. These are not contradictions, but points where clarity is needed.

Divergence among disciplines on the use of these terms makes reaching consensus complicated. In particular, the concept of “environment” is inconsistently defined and operationalised (e.g. Canali and Leonelli, 2022; Malsch *et al*., 2024). There are also many ambiguities in the use of “environment” that we wish to avoid. For example, humans and other animals are “environments” for microbial life (microbiome One Health is a burgeoning field) (Tomasulo *et al*., 2024). Also, we can speak about “social environments” to describe the way infrastructures, institutions, and interactions with other humans condition a given person’s experience and possibilities for action. Indeed, sometimes “environment” is used *because* of its ambiguity. We also sometimes see modifications to the term “ecosystem”, such as “anthropo-ecosystem” or “socio-ecosystem”.

In the final analysis, “ecosystem” is a more relevant, encompassing concept than “environment”, and this is of key importance. Although “environment” is the more common term, policy makers will already be familiar with “ecosystem”, not least as it features in the OHHLEP definition.

For pragmatic reasons, there are circumstances where it nevertheless makes sense to use “environment” (or “nature”) instead of “ecosystem”, while recognising that they are not ideal terms. Entities like human-made structures and geological features are more readily understood as “environments” since they are places, even though they might well be part of ecosystems: consider how chemicals in manufactured homes and furnishings can cause human and animal illness, or how different microclimates on a mountainside allow different plants and animals to thrive and fulfil roles in food chains. As noted above, we navigate between speaking carefully for precise thought and speaking pragmatically in familiar language to communicate and persuade – indeed; this navigation is necessary to implement One Health.

The underlying One Health philosophy and movement away from anthropocentrism means that we try to use the expansive concept of “ecosystem” when possible, instead of the common “three components” framing. This emphasises the complex web of relationships that exist between different kinds of living and non-living entities. It encourages a more holistic approach to understanding and managing ecosystems and by extension the components of, or contributing factors within, ecosystems (including humans). We take inspiration from posthumanist philosophy and more radical framings, such as those of biologist and feminist science studies scholar Donna Haraway, whose philosophy of what the world is and how it can be known emphasises *sympoiesis* (becoming together) and how humans are in constant collaboration with the “more-than-human” (Haraway, 2016). We emphasise that such an “ecosystem philosophy” is original to many Indigenous perspectives around the world, and that, going forward, One Health theory will benefit enormously from sustained and receptive conversations with these perspectives (e.g. LaDuke, 1999).

### What Do We Mean By “Health”?

Defining One Health calls for a secondary definition of “health” itself, allowing us to be clear about the ends, and not just the process by which we hope to achieve them. Although we cannot devote much space to this important issue, we work from the World Health Organization definition of health as a state of complete physical, mental, social, emotional, and spiritual wellbeing, not merely the absence of disease or infirmity. This definition is included in the Sundsvall Statement on Supportive Environments for Health (Buse *et al*., 2023).

We interpret this definition as consistent across humans, non-human animals, and ecosystems. We recognise that health needs may sometimes conflict, thereby raising ethical concerns and unresolved issues related to equity between sectors (e.g. Coghlan *et al*., 2021; Zinsstag *et al*., 2023). For example, One Health ethics will need to develop ways to negotiate consensus between diverging norms and strategies for livelihood, animal welfare, ecosystem protection, and health of all species (LaDuke, 1999; MacKillop *et al*., 2023). Mindful of this, we emphasise that a systems approach is necessary to manage the complexities and that integrating low- and middle-income countries and Indigenous perspectives in defining and operationalising health is essential.

### “Integrated” and “Unifying”

The way we have defined “ecosystem” emphasises unification – that is, wholeness – instead of the integration of essentially separate entities. However, we also recognise that it makes pragmatic sense to identify components of the whole and work to integrate them in the way they are studied and managed, given the legacies of having separated humans, animals, and environments in science, policy, infrastructure, and so on.

### “Balanced” and “Optimised”

The way we have defined “ecosystem” moves One Health away from the presumption of humans at the top of a pyramid of importance. This is key to operationalising One Health. Ideally, resources would be more equally distributed among initiatives and institutions for the health of humans, animals, and environments. For example, the environment has been called “the neglected component” of the triad and consequently described as a “threat multiplier” (Essack, 2018). However, we recognise that an ecosystem has many components (more than three) that are not separable and should not necessarily be considered equal in order to achieve a more holistic, dynamic version of balance; this is a complex issue for future attention.

In general, we understand “optimisation” to mean the most efficient route to the best possible (ecosystemic) health. Currently, what it means to “optimise” and “operationalise” something is inherently capitalist (Szocik, 2024). However, economic growth is often at odds with healthy ecosystems (Raworth, 2017; Stuart *et al*., 2020; Asdal *et al*., 2023). In our view, a (de)growth or wellbeing economy is integral to a One Health approach. Furthermore, we must operate from the premise that health is an asset and not a cost.

Yet we recognise the short-term importance of seeking optimisation in the shape of “incremental benefits”, meaning added value through more economically and logistically efficient ways of safeguarding and promoting health (i.e. cost minimisation).

### “Closely Linked” and “Interdependent”

Close linkage, or “interconnection”, is a way of integrating entities presumed to be distinct. Interconnection implies causal interaction (e.g. that phytopathology and the related use of pesticides may result in biodiversity loss, in turn, favouring the emergence of animal and human disease). “Interdependence” is more extreme because it implies that humans, animals, plants, microbes, soil, climates, and so on are not separable and have a closer relationship of mutual constitution (e.g. an animal and its microbiome). We understand these two concepts as existing on a spectrum. The way we define and understand an “ecosystem” includes both, although it is more fundamentally aligned with interdependence.

Interconnection “entails that the universe is organised into discrete units, which interact with one another and, consequently … affirms a particular ontology of the individual as internally structured and autonomously motivated” (Beever and Morar, 2019). The ontology of the individual is deeply embedded in conventional ways of approaching health. Our view of an ecosystem is more aligned with process philosophy, which speaks about the biological world as a web of mutual dependencies and “an ecological view of reality” as a “highly integrated and dynamic pattern of interdependent events” (Barbour, 1997). Such interdependence, however, could have some radical ethical implications. For example, Sironi and colleagues argue that it implies ecological egalitarianism, “that we should not give precedence to any element of the natural world”, including humans (Sironi *et al*., 2022). This is in line with the ultimate goals of unifying, balancing, and optimising, but may be difficult to put into practice as it conflicts with conventional anthropocentric ethical beliefs and current infrastructures.

Interconnection has been called “a prudential approach” (Sironi *et al*., 2022) or a “moderate approach” (Beever and Morar, 2019), while interdependence has been called “a radical approach” (Sironi *et al*., 2022) and “a strong approach” (Beever and Morar, 2019). We recognise that sometimes it will be more realistic to emphasise interconnection than interdependence. Moving between these moderate and strong approaches is needed to interact pragmatically with different stakeholders and move towards an ecological paradigm. This prudent approach is currently necessary given the lack of institutions designed around interdependence, a situation we anticipate will gradually shift.

### Synergies With Other Terms

Other terms have circulated alongside One Health to describe similar approaches, including “Planetary Health” and “Eco Health”. Discussions about food systems and sustainability are also adjacent to One Health’s approach and goals. We see these not as competing, but synergistic and complementary. Attempts have been made to identify similarities, for example with “sustainable health” (Wanyenze *et al*., 2023) and “global and planetary health” (Correia *et al*., 2021). Complementary operationalising theories have developed, such as “One Health Systems”, which aim to avoid blind spots in the approaches adopted by acknowledging “building blocks” (Ferrinho *et al*., 2023).

Given that planetary health (also called “One Planet” and “Healthy Planet”) has attracted recent attention and investment, more must be said about synergies with this approach. Planetary health discourses tend to prioritise and start from human health (Zinsstag *et al*., 2023), which is counter to our aims. The report of the Rockefeller Foundation-Lancet Commission for Planetary Health states that “Put simply, planetary health is the health *of human civilisation* and the state of the natural systems on which it depends” (Whitmee *et al*., 2015, italics added). Although oriented towards a somewhat different audience (one that often features physicians), common ground may exist between the planetary health paradigm and One Health, given our earlier explanation that the planet can be considered an ecosystem.

Planetary health uses key performance indicators to measure where human existence itself is called into question based on exceeding planetary boundaries. We hope to integrate and shift this view on planetary resources – which describe the planet’s basic habitability for humans – into an expansive, non-anthropocentric definition of health that recognises that the planetary ecosystem is both essential for human health and wellbeing, and also of value in its own right. In a sign that such an expansion is already taking place, social justice was recently incorporated into Planetary Health definitions, a promising move that nonetheless urges us to think carefully about the potentially totalitarian implications of operationalising health at the planetary level (Roth and Valentinov, 2023). The added value of a One Health approach has generally been cast in terms of economics, yet as we have touched on in this article, it offers additional benefits specifically related to ethics.

We want to be careful not to trivialise the issues at stake by becoming stuck in semantic differences. Rather, we acknowledge these other terms as instances of similarly concerned parties pulling on the same rope from a slightly different direction. As noted above, One Health approaches should be tailored to the relevant audience to maximise receptivity and effectiveness. Existing frameworks for integrating diverse perspectives could be usefully adapted; for example, the concept of “Two-Eyed Seeing” which advocates simultaneously considering Indigenous and Western perspectives (Wright *et al*., 2019).

## Conclusion

One Health is a powerful paradigm-shifting approach that promises not only to improve health but also to tackle the most pressing issues of our time. To realise its potential, we must take pragmatic steps from within imperfect systems. This reflection has set out the all-encompassing scope of One Health, articulated clear definitions of the terms we use and their theoretical implications, and argued for flexibility in the approach so that we can achieve the potential of One Health in efficient and collaborative ways. We note that One Health ethics will require further consideration in the future, given its paradigm-shifting character.

With all the above under consideration, we propose this adaptation of the OHHLEP 2022 definition:

*One Health is an integrated, unifying approach that aims to sustainably balance and optimise the health of ecosystems, which encompass people, domestic and wild animals, and all aspects of their environments. It recognises that the health of humans, animals, and their environments are closely linked and interdependent*.

The OHHLEP definition has found consensus in many existing documents. We hope our analytical clarifications assist it in being relevant and useful in practice.

## Data Availability

As a conceptual paper, no datasets are relevant.

## One Health impact statement

Defining a concept is an opportunity to work through contradictions and imprecisions that can hinder practical application. The concept of One Health is transformative and complex. While substantial attention has been given to defining it, ambiguities remain. In addition to possible conflicts and frictions in applying One Health principles, these ambiguities have political and ethical stakes. Fully integrating the third principle of environmental health and granting it as much attention and importance as human and animal health is a challenge with practical and ethical/political dimensions. The conceptual work in this article will help implement One Health and realise its added value potential. It is transdisciplinary in that it was developed by a highly interdisciplinary group of scholars, spanning humanities/social sciences (philosophy, anthropology, legal studies, public health), and biological sciences (veterinary medicine, parasitology), all of whom are engaged with clinical practice, policy making, and/or community-based work.
